# The Current Understanding of Autophagy in Nanomaterial Toxicity and Its Implementation in Safety Assessment-Related Alternative Testing Strategies

**DOI:** 10.3390/ijms21072387

**Published:** 2020-03-30

**Authors:** Rong-Jane Chen, Yu-Ying Chen, Mei-Yi Liao, Yu-Hsuan Lee, Zi-Yu Chen, Shian-Jang Yan, Ya-Ling Yeh, Li-Xing Yang, Yen-Ling Lee, Yuan-Hua Wu, Ying-Jan Wang

**Affiliations:** 1 Department of Food Safety/Hygiene and Risk Management, College of Medicine, National Cheng Kung University, Tainan 704, Taiwan; janekhc@gmail.com; 2 Department of Environmental and Occupational Health, College of Medicine, National Cheng Kung University, Tainan 704, Taiwan; 101312123@gms.tcu.edu.tw (Y.-Y.C.); q781001@gmail.com (Z.-Y.C.); linn7627@hotmail.com (Y.-L.Y.); 3 Department of Applied Chemistry, National Pingtung University, Pingtung 900, Taiwan; myliao@mail.nptu.edu.tw; 4 Department of Cosmeceutics, China Medical University, Taichung 651, Taiwan; yhlee@mail.cmu.edu.tw; 5 Department of Physiology, College of Medicine, National Cheng Kung University, Tainan 701, Taiwan; johnyan@mail.ncku.edu.tw; 6 Institute of Oral Medicine and Department of Stomatology, College of Medicine, National Cheng Kung University Hospital, National Cheng Kung University, Tainan 701, Taiwan; fingerzoo@gmail.com; 7 Department of Hematology/Oncology, Tainan Hospital of Health and Welfare, Tainan 700, Taiwan; yenpig8291@gmail.com; 8 Department of Radiation Oncology, National Cheng Kung University Hospital, College of Medicine, National Cheng Kung University, Tainan 704, Taiwan; 9 Department of Medical Research, China Medical University Hospital, China Medical University, Taichung 404, Taiwan

**Keywords:** nanomaterials, autophagy, alternative testing strategy, high throughput screening, tiered testing strategy, *C. elegans*, zebrafish and Drosophila models

## Abstract

Nanotechnology has rapidly promoted the development of a new generation of industrial and commercial products; however, it has also raised some concerns about human health and safety. To evaluate the toxicity of the great diversity of nanomaterials (NMs) in the traditional manner, a tremendous number of safety assessments and a very large number of animals would be required. For this reason, it is necessary to consider the use of alternative testing strategies or methods that reduce, refine, or replace (3Rs) the use of animals for assessing the toxicity of NMs. Autophagy is considered an early indicator of NM interactions with cells and has been recently recognized as an important form of cell death in nanoparticle-induced toxicity. Impairment of autophagy is related to the accelerated pathogenesis of diseases. By using mechanism-based high-throughput screening in vitro, we can predict the NMs that may lead to the generation of disease outcomes in vivo. Thus, a tiered testing strategy is suggested that includes a set of standardized assays in relevant human cell lines followed by critical validation studies carried out in animals or whole organism models such as *C. elegans* (Caenorhabditis elegans), zebrafish (Danio rerio), and Drosophila (Drosophila melanogaster)for improved screening of NM safety. A thorough understanding of the mechanisms by which NMs perturb biological systems, including autophagy induction, is critical for a more comprehensive elucidation of nanotoxicity. A more profound understanding of toxicity mechanisms will also facilitate the development of prevention and intervention policies against adverse outcomes induced by NMs. The development of a tiered testing strategy for NM hazard assessment not only promotes a more widespread adoption of non-rodent or 3R principles but also makes nanotoxicology testing more ethical, relevant, and cost- and time-efficient.

## 1. Introduction

Nanomaterials (NMs) are defined as having at least one dimension that is 1–100 nm in diameter [1] and unique properties; for example, they can change reactivity, optical characteristics, or conductivity, thereby enabling novel applications. Furthermore, particle properties can be modified to promote different applications, resulting in consumer benefits, particularly in medical and industrial applications [2]. In recent years, nanotechnology has rapidly been promoted in the development of a new generation of industrial and commercial products. It has been estimated that the nanoproduct demands in medicine and pharmaceuticals, and especially the cosmetics industry, are expected to rise by over 17% each year and at a much higher rate in the food industry [3,4]. However, the application of nanotechnology has also raised some concerns about human health and safety. In some cases, nanomaterials present unexpected risks to both humans and the environment. Regulatory authorities in the European Union, United States, and Asian countries carefully observe developments in nanotechnology, trying to find a balance between consumer safety and the interests of the industry [5]. In addition, several international planning activities have been proposed or performed with the expectation that significant advances will be made in understanding the potential hazards triggered by nanomaterial exposure in both occupational and consumer environments [2].

Assessments of the potential hazards associated with nanotechnology have been emerging, but substantial challenges remain because all of the different nanoparticle (NP) types cannot be effectively evaluated for safety and environmental effects in a timely manner [2]. Identification of the physicochemical properties of nanomaterials that confer toxicity is a core component of toxicity studies. To evaluate the toxicity of the great diversity of NMs, a tremendous number of safety assessments would need to be conducted. It was estimated that, in 2009, a complete toxicity evaluation of all the nanomaterial on the market using traditional animal approaches would cost more than 1 billion US dollars, take at least 50 years, and require a very large number of animals [6,7]. Whether animals can be used to predict human response to toxicant exposure is still under debate, attributing to data gap between human and animal studies. Thus, there is a need for developing and using human-cell-based methods that generate human-relevant mechanistic data that are not necessarily obtainable from traditional animal studies conducted by vertebrate animals [8,9,10]. Furthermore, there are government regulations that have resulted in an enhanced need for alternative methods, such as the E.U. Cosmetics Directive that prohibits the testing of cosmetics products on animals in the European Union (EU Regulation 1223/2009). For all these reasons, it is necessary to consider the use of alternative testing strategies or methods that reduce, refine, or replace (3Rs) the use of animals for assessing the toxicity of nanomaterials [10].

Alternative testing strategies are commonly used to assess the safety of chemicals, and many of these strategies have been evaluated for their applicability to the testing of nanomaterials. A single alternative testing method may contribute to basic mechanistic or toxicity knowledge but may not be sufficient for use in hazard assessment. However, incorporating multiple alternative testing methods into alternative testing strategies will provide an understanding of the behavior and toxicity of nanomaterials in humans and the environment [10,11]. In vitro testing was proposed as the principal approach with the support of in vivo assays to fill knowledge gaps, including tests conducted in non-mammalian species such as *C. elegans*, Drosophila, and zebrafish, or genetically engineered animal models. These tools are being used to identify responses in cells exposed to chemicals expected to result in toxic effects [9,12]. Well-designed alternative testing strategies will not only allow for the prioritization of nanomaterials for further testing but can also assist in the prediction of risk to human beings and the environment.

Autophagy is a catabolic mechanism that is evolutionarily conserved from yeast to mammals. The autophagy pathway first described by Christian De Duve in 1963 [13] is a ubiquitous process that involves the degradation of cytoplasmic components and cytoplasm organelles, that degrade through the lysosomal pathway, and is distinct from other degradative pathways, such as proteasomal degradation [14]. When energy is limiting (ATP shortage), AMP kinase (AMPK) is activated, which can drive autophagy. Similarly, deprivation of growth factors or amino acids leads to the inhibition of TORC1, which is a repressor of conventional autophagy [15]. The inability to regulate autophagy is associated with aging, neurodegeneration, and a variety of diseases, including cancer, type 2 diabetes, and atherosclerosis [16]. Autophagy was recently recognized as an important form of cell death in various types of nanoparticle-induced toxicity, but the details of the underlying mechanisms are still unclear. A thorough understanding of the cellular and molecular mechanisms of nanoparticle-triggered toxicity is critical for a more comprehensive elucidation of nanotoxicity [17]. Our recent work provides the first demonstration that autophagy activated by silver nanoparticles (AgNPs) in normal cells fails to trigger lysosomal degradation pathway and led to a toxicity phenomenon called defective autophagic flux or autophagy dysfunction, which is relevant to the accelerated cellular pathogenesis of diseases [18,19,20]. The toxic effects induced by AgNPs and some of the metal oxide NPs, such as ZnONPs, have been shown, either in vitro or in vivo, to be quite similar in terms of cytotoxicity, genotoxicity, hematotoxicity, immunotoxicity, hepatotoxicity, and embryotoxicity [19,21]. A more profound understanding of these toxicity mechanisms will facilitate the development of prevention and intervention policies against adverse outcomes induced by metal and metal oxide nanomaterials. Knowledge derived from the cellular and molecular processes underlying nanomaterial-induced toxic effects may also facilitate the establishment of the scientific foundations of nanomaterial risk assessment. Therefore, an overview of current findings regarding the mediation of autophagy triggered by NPs both in vitro and in vivo will shed light on the pivotal role of autophagy in nanomaterial toxicity and the useful implementation of autophagy in safety assessments conducted through alternative testing strategies.

## 2. Alternative Testing Strategy for Nanomaterial Safety Assessments

### 2.1. Approach towards the Use of Alternatives to Testing on Animals

The “3 Rs”, standing for reduction, refinement, or replacement, is a strategy being applied to the use of laboratory animals through implementation of different methods and alternative organisms to provide integrated approaches that could provide insight into the minimal use of animals in scientific experiments [22]. In 2007, the US National Research Council (NRC) published a report entitled “Toxicity Testing in the 21st Century (TT21C): A Vision and a Strategy”, putting forward a long-term strategy taking advantage of newly developed technologies to enhance the efficiency of the toxicity testing of chemicals to which human beings may be exposed. The important parts of this strategy are the increased use of high-throughput in vitro test systems and methods in computational toxicology for the purpose of reducing the reliance on time-consuming and costly toxicological studies using experimental animals. This vision has received international support and has provided a blueprint for implementing change in toxicological science [9,23]. In 2011, the European Union launched SEURAT-1, the first execution phase of “Safety Evaluation Ultimately Replacing Animal Testing (SEURAT)”, with the ultimate goals of the future implementation of mechanism-based, integrated toxicity testing strategies into modern safety assessment approaches [24]. In addition, promotion of non-animal approaches is also among the objectives of the REACH (Registration, Evaluation, Authorization, and Restriction of Chemicals), CLP (Classification, Labeling, and Packaging) and BPR (Biocidal Products Regulations) initiatives, which are based on the 3Rs principle of animal use for testing [25]. Through the abovementioned efforts, many government agencies within the United States, European Union, and other international bodies are beginning to incorporate the new approach methodologies envisioned in the original TT21C vision into regulatory practice [23].

Current experimental toxicology approaches are being promoted rapidly by the incorporation of novel techniques and methods that provide a much more in-depth view into the mechanisms of potential adverse effects of chemical exposure to human health [26]. For example, the basal cytotoxicity level determined by in vitro cytotoxicity assays is considered a key factor in many prevalent toxicological modes of action associated with the mechanisms of organ failure, including disruption of cell membrane structure and/or function, disturbance of protein turnover, inhibition of mitochondrial function, and disruption of metabolism and energy production [27,28,29,30]. In 2017, Vinken and Blaauboer proposed three consecutive steps, including initial cell injury, mitochondrial dysfunction, and cell death, as the adverse outcome pathway (AOP) framework for measuring basal cytotoxicity. The outcome of basal cytotoxicity assessment could serve as the first step of a tiered strategy aimed at evaluating the toxicity of new chemicals, and then, more specific types of toxicity could be evaluated in a second step [29]. The data from various in vitro assays are useful for both increasing confidence in hazard and risk decisions and enabling better, faster, and less expensive assessments of a large number of chemicals, mixtures, and complex products.

### 2.2. Alternative Testing Strategy for Nanomaterials

The lack of availability of regulatory guidelines for the safety assessment of nanomaterials is a major problem. In general, the Organization for Economic Cooperation and Development (OECD) safety assessment of traditional chemicals is suitable for the nanomaterial safety assessment but needs adaptation. Owing to the unique physical/chemical properties of nanomaterials, the original OECD guidelines need to be further adjusted and improved [7]. Our understanding of the mechanisms of nanomaterial-induced toxicity is insufficient for drawing a general consensus and/or conclusion on the toxicity of nanomaterials [31]. To assess nanomaterial hazards, reliable screening approaches are required to test the basic materials as well as the nano-enabled products. The European Union launched the FP7 NanoTEST project (www.nanotest-fp7.eu) to provide testing strategies for the hazard identification and risk assessment of nanomaterials and to propose recommendations for evaluating the potential risks of newly designed nanomaterials [11]. However, the knowledge gaps of nanomaterial behavior, such as its transformation and fate in biological systems, make it difficult to perform adequate hazard assessment. Thus, a better understanding of nanomaterials with cells, tissues and organs for addressing critical issues related to toxicity testing, especially with respect to alternatives to testing animals, is needed [11]. How nanomaterials interact with biological systems has become an important and complex issue in terms of both the research and regulatory options. When nanomaterials encounter biomolecules or cells, their physicochemical properties have a major impact on the degree to which the material adversely perturbs biological systems [32,33]. Nano-bio interactions may also be affected by the properties of different cell types, the biological environment, and the assay methods applied, making the issues more complicated. A thorough understanding of the mechanisms regarding nanomaterials-induced perturbation in biological systems such as autophagy induction is critical for a more comprehensive elucidation of nanotoxicity [33,34]. 

In vitro studies are mainly performed on cell lines, which are transformed and immortalized cells to escape normal cellular senescence. These well-established cell lines are cheap, readily available, and easy to passage because of enhanced proliferation ability [35]. On the contrary, primary cells are isolated from tissue without any modification and have similar characteristics as the original donor tissue, so more and more people are considering the use of primary cells in in vitro studies to establish more biologically representative models [35]. Several research groups have compared the effects of NPs exposure between primary cells and immortalized cell lines representing the same tissue, and claimed that immortalized cell lines were more sensitive to NPs toxicity than primary cells [35]. However, in some cases, since immortalized cell lines are de-differentiated in culture, they may not respond to certain cell reactions [36,37]. Therefore, the nanotoxicity results of in vitro studies are often not very relevant to the results of in vivo studies. However, Drasler et al. suggest that immortalized cell lines are suitable for the first stage of nanosafety assessment, because they can provide greater comparability and reproducibility for interlaboratory comparisons of the same NM type or between different NMs. In terms of higher tier evaluation, using primary cells to better understand the NM mechanism in the human body is preferred, as they can more closely mimic in vivo conditions [36].

A strategy for in vitro toxicity testing requires a series of tests addressing and covering different mechanisms important toxicity endpoints. Thus, to identify relevant short-term hazard models, several different outcomes, such as cell viability, oxidative stress, genotoxicity, the proinflammatory response, immunotoxicity, cell uptake, and transport, are conducted [38]. By using mechanism-based high-throughput screening in vitro, [7] we can predict the nanomaterials that may lead to the generation of target organ toxicity in vivo. Additional in vivo studies are used to validate and improve the in vitro high-throughput screening process and to establish structure–function relationships that enable hazard ranking and modeling by an appropriate combination of in vitro and in vivo testing [38,39]. Thus, a tiered testing strategy was suggested that includes a set of standardized cytotoxicity assays in relevant human cell lines followed by critical validation studies carried out in animals or whole organism models such as *C. elegans*, zebrafish, or Drosophila for improved screening of nanomaterial safety [31]. Zebrafish have been deemed acceptable by regulatory agencies for use in chemical safety assessments for evaluating developmental toxicity and are now regularly accepted models in biomedical research, providing strong foundations for their use in nanotoxicology [39]. The development of a tiered testing strategy for nanomaterial hazard assessment not only promotes the widespread adoption of non-rodent and the 3R principles but also makes nanotoxicology testing more ethical, relevant, and cost- and time-efficient [7].

## 3. Updated Nanotoxicology Knowledge Regarding Autophagy Dysfunction

### 3.1. Autophagy and Autophagy-Induced Cell Death (ACD)

Dysregulated cell death is a common feature of many human diseases, and the complex mechanisms and pathways that control cell death are becoming increasingly understood. It is now clear that different cell death pathways have a critical role in multiple diseases (such as Crohn’s, Parkinson’s, and Alzheimer’s diseases) [16]. Currently, there are currently three common well known cell death pathways, including apoptosis (type I programmed cell death), necrosis, and the so call autophagic cell death (also referred to as type II programmed cell death) [40]. The association between autophagy and cell death has been known for many years. Originally, based on morphology, autophagic structures were observed in dying cells and distinguished autophagic from apoptotic cell death. Under stress conditions, autophagy is initially induced as an early pro-survival response in the cell, but accumulated autophagy-related substances contributes to autophagic cell death (ACD) [16]. ACD is characterized by the large-scale autophagosomes sequestration in the cytoplasm, giving the cell a characteristic vacuolated shape [16]. Autophagosomes can be identified by transmission electron microscopy as double-membraned vesicles that contain cytosol or cytoplasmic organelles such as mitochondria or the endoplasmic reticulum. Numerous reports, particularly from model systems, provide support for a direct role of autophagy in cell death in context-dependent settings [16,40]. Despite this evidence, our understanding of the mechanism by which autophagy contributes to cell death is not clear. The cross talk between autophagy and other cell death pathway components suggests that the role of autophagy may be context-specific, and understanding the molecular nature of these relationships will aid in understanding the role of ACD.

### 3.2. Autophagy Dysfunction as a Cell Toxicity Mechanism

Autophagy dysfunction is defined as massive autophagy induction or a blockade of autophagic flux. It is recognized as a potential mechanism of cell death, resulting in either apoptosis or ACD [40]. Previous report proposed that autophagy proteins LC3-II and ATG5 may directly activate caspase, causing cell death through its interactions with Fas and Fas-associated proteins with a death domain (FADD) [41]. In addition, defective autophagy can lead to cancer development, possibly by accumulation of damaged organelles, such as mitochondria, that can induce oxidative stress, inflammation, and DNA damage. The pro-autophagy gene beclin-1 is commonly deleted in several types of cancer, such like breast, ovarian, and prostate cancer, suggesting a tumor suppressor function in the autophagy pathway. Furthermore, beclin-1-knockout mice exhibit enhanced susceptibility to cancer development [42]. Defective autophagy has also been associated with many diseases, such as Crohn’s, Parkinson’s, and Alzheimer’s diseases and may play a role in disease development [43]. In the case of Crohn’s disease, disruption of autophagy during immune and inflammation responses may be involved in disease progression. In Parkinson’s and Alzheimer’s disease, blockade of autophagy-mediated elimination of amyloid beta and alpha synuclein proteins or damaged mitochondria may be involved [43].

Disruption of lysosomal trafficking is a major mechanism for blocking autophagic flux, which results in the accumulation of autophagic and lysosomal vacuoles. There are several possible mechanisms by which NPs might disrupt autophagy and lysosomal trafficking. For example, lysosomal overload by particulates has been proposed as a mechanism by which cigarette smoke blocks autophagic flux in alveolar macrophages [44]. One common form of lysosomal dysfunction that has been associated with nanomaterial treatments is increased lysosomal membrane permeabilization (LMP). Proton pump inhibitors, such as bafilomycin A1, that block autophagic flux predispose cells to LMP, triggering apoptosis through the LMP-induced release of pro-apoptosis mediators such as cathepsin [45]. LMP is a recognized cell death mechanism that can result in mitochondrial membrane permeabilization through several mechanisms, including lysosomal-iron mediated oxidative stress and the release of cathepsin or other lysosomal associated hydrolases [45]. As lysosomal dysfunction has been involved in disease pathogenesis, the association of nanoparticle exposure and lysosomal dysfunction may have relevance to nanomaterial-induced toxicity levels, especially chronic toxicity. Since lysosomal degradation pathways play vital roles in cellular homeostasis, lysosomal dysfunction has been associated with several diseases, termed lysosomal storage disorders [43,46]. Many types of lysosomal perturbations are also associated with autophagy dysfunction, blocking autophagosomes and lysosome fusion and promoting the accumulation of autophagosomes and other autophagy-related substrates (e.g., ubiquitinated protein aggregates) [45]. Autophagy dysfunction can result from lysosomal overload, which prevents autophagosome–lysosome fusion. Similar to those causing lysosomal dysfunction, perturbations in the autophagy pathway have been linked to a variety of diseases [47].

### 3.3. Autophagy Dysfunction Induced by Nanomaterials

**Figure 1:**
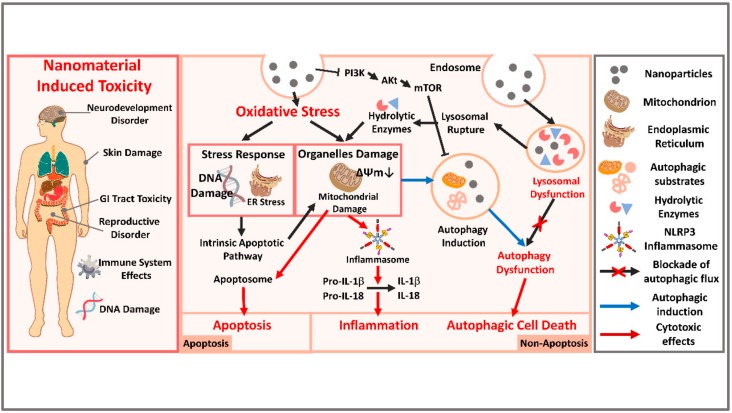
An overview of the mechanism of nanomaterial-induced autophagy-related toxicity and subsequent systematic potential toxic effects. The overall toxicity caused by nanomaterials includes neurodevelopmental disorders, skin damage, gastrointestinal tract toxicity, reproductive disorders, immune system effects, DNA damage, and so on. One of the major forms of toxicity caused by nanomaterials is the oxidative stress induced by ROS and resulting in ER stress and mitochondria and DNA damage. The stress response and organelle damage can eventually induce apoptotic cell death. ROS production results in mitochondrial damage that activates the NLRP3 inflammasome and cellular inflammation. Another major implication for nanomaterial-induced toxicity is autophagy dysfunction. Nanomaterial-induced autophagy and lysosomal dysfunction are displayed as blue arrows in the figure. The initiation step of autophagy is induced by the accumulation of nanomaterials in autophagosomes and blocked vesicle trafficking or by the inhibition of the PI3K/Akt/mTOR pathways. The second step of autophagic toxicity can be induced by overloading of nanomaterials in the lysosomes, leading to damage to the organelle compartments, lysosomal membrane permeabilization (LMP), and release of hydrolytic enzymes. The damaged lysosomes also cause blocked autophagosome-lysosome fusion, eventually leading to autophagic cell death. Altogether, stress responses and organelle damage may synergistically promote cell death, including that caused by apoptosis activation, NLRP3 inflammasome activation, or autophagy.

Autophagy disturbances have been reported consistently across several types of nanomaterials and biological models. There are several plausible pathways of nanomaterials-induced autophagy dysfunction (Figure 1). Studies have attempted to illuminate the importance of cellular nanoparticle internalization pathways in nanotoxicity. For example, researchers found that intracellular AgNPs possess limited or no cytotoxic effects when intracellularized AgNPs were shown to exhibit free random Brownian motions within the cytosol rather than accumulate in lysosomes [19]. By contrast, they identified that AgNPs actively internalized via endocytosis were predominantly trafficked within the endo-lysosomal compartments and were obviously toxic to cells [19]. For instance, nanomaterials may induce autophagy through an oxidative stress mechanism [48], such as by the accumulation of reactive oxygen species (ROS), damaged proteins and the endoplasmic reticulum Stress (ER stress) or mitochondrial damage [48]. The involvement of oxidative stress in the induction of autophagy by nanomaterials is supported by a study in which silica nanoparticles induced autophagy in human endothelial cells via reactive oxygen species-mediated MAPK/Bcl-2 and PI3K/Akt/mTOR signaling, which was suppressed by cell exposure to the antioxidant N-acetyl-L-cysteine [49]. Alternatively, nanomaterials may directly affect autophagy-dependent signaling pathways or gene/protein expression. It is also likely that autophagy induction by nanomaterials is the result of an attempt by the cell to degrade what is perceived as foreign or aberrant, similar to cellular action against bacteria and other pathogens. As discussed above, nanoparticles are commonly observed within the autophagosomes compartment, suggesting that the activation of autophagy is triggered by the attempt to sequester and degrade materials that enter into the cytoplasm [50,51]. Cytoplasmic nanoparticles undergo ubiquitination and are co-localized with polyubiquitin complexes that are then translocated to the autophagosomes by p62 [52]. The induction of autophagy has also been observed following treatment with different types of nanoparticles, such as silica nanoparticles, silver nanoparticles, and others [50,53]. Autophagy is activated in human cancer cells by zinc oxide nanoparticles, which are currently under development for use in enhancing tumor chemotherapy and overcoming drug resistance [54]. Quantum dots are also currently under development for use in a broad range of biomedical imaging applications and have been shown to induce lysosome-dependent autophagy activation, ROS production, and toxicity in human hepatocytes [55]. As explained above, there is little evidence of autophagy as an actual effector of cell death, and the cytotoxicity resulting from blocking autophagic pro-survival mechanisms appears to be the more likely effect of nanomaterial exposure. Since blocking autophagic flux and autophagy induction can both lead to autophagosome accumulation [16], the mechanism by which nanomaterials induce autophagosome accumulation is unclear in many cases. Nonetheless, the disruption or blockade of autophagic flux is often observed in cells exposed to nanomaterials.

Many studies have revealed the connection between nanomaterial-induced autophagy dysfunction and mitochondrial damage [56,57]. Disruption of the autophagy pathway by gene knockout has also been associated with the accumulation of dysfunctional mitochondria and ROS, thus providing a potential link between nanomaterial-induced autophagy blockade and oxidative stress (Figure 1) [58]. Nanomaterial-induced autophagy blockade may also be a mechanism by which nanomaterials induce inflammation, due to the fact that autophagy plays an important role in negatively regulating the NLRP3 inflammasome [53]. Blockade of autophagic flux may result in mitochondrial dysfunction through prevention of the removal of the damaged mitochondria, which are normally degraded in the normal autophagy pathway [57]. Consistent with autophagy being assumed to be a mechanism to diminish damaged mitochondria, there is also evidence that mitochondrial depolarization actually led to autophagy induction [57]. Thus, it is conceivable that nanoparticles might be expected to result from a combination of autophagy induction and autophagy blockade, which may be triggered with an increased number of depolarized, dysfunctional mitochondria that cannot be cleared because of impaired autophagic flux. Another major mechanism of nanoparticle-induced autophagy pathway dysfunction is lysosomal dysfunction. Many studies have observed nanomaterial-induced lysosomal dysfunction (Figure 1) [59,60]. There are many plausible explanations for nanoparticle-induced lysosomal dysfunction, including inhibited enzyme ability and bio-persistence [55]. The “proton sponge” hypothesis for cationic nanoparticles is a well-known theory of nanoparticle-induced lysosomal dysfunction, which involves osmotic swelling and membrane rupture [61]. Another direct mechanism that might account for nanoparticle-induced lysosomal dysfunction is the generation of ROS [62]. As many nanoparticles can induce ROS, the oxidative stress model is by far the most accepted theory of nanoparticle-induced toxicity [48].

## 4. In Vivo Model Systems for the Detection of Nanomaterial Toxicity and Autophagy

### 4.1. C. elegans Model

*Caenorhabditis elegans* (*C. elegans*) is a well-established small nematode model organism that has been used since the 1970s [63]. Unlike the traditional toxicity cell culture testing systems, *C. elegans* provide data from a whole animal with complete and metabolically active digestive, reproductive, endocrine, sensory, and neuromuscular systems [64]. Indeed, *C. elegans* research has been proved to be essential in the clarification of several basic aspects of biology, including apoptosis, autophagy, RNA interference, and miRNA function. It has also been demonstrated that the results conducted in *C. elegans* have consistently shown good correlation with rodent oral LD50 ranking [65]. Due to the rapid needs of nanotechnology assessment, especially at the environmental exposure and risk assessment, *C. elegans*, as a complete model organism, has become an important in vivo alternative assay system to assess the risk of NPs [66]. The interaction between NPs and *C. elegans* can be used for providing the toxicity outcome of NPs in a multicellular organism. Recently, *C. elegans* has been used in acute, prolonged, and chronic exposure by using oral treatment, topical applications, or microinjection to particular organs. In addition, *C. elegans* as a whole organism, is able to provide different toxicity endpoints, such as immunotoxicity, neurotoxicity, reproductive toxicity, and genotoxicity [66]. The assessment of the interaction between NPs and *C. elegans* provides information of the in vivo behavior and biocompatibility in a multicellular organism of some NPs for evaluating their fate and toxicity [67].

Regarding the nanomaterial-induced autophagy, *C. elegans* has been employed to identify and characterize the autophagy-regulating mechanism, and 139 conserved genes that regulate autophagy activity are identified, offering a framework for thorough dissection of the autophagy process [68]. Studies show that treatment of quantum dots (QDs), gold nanoparticles (AuNPs), and carbon dots (CDs) are able to induce massive autophagosome formation, autophagy related gene upregulation, and autophagy substrate degradation in cultured HeLa cells and in live *C. elegans* [64]. Due to the small size of *C. elegans*, it is very easy to track the autophagosome formation in real-time by simply using the fluorescence microscopy [69], making *C. elegans* a suitable model organism for the alternative nanotoxicity approach and providing great connection between in vitro and in vivo toxicity [69].

### 4.2. Zebrafish Model

The extensive applications of nanoparticles in various aspects of daily life, such as the healthcare and industrial sectors, have increased the concern of their impact on human health and the environment [70,71]. Several models have been applied to investigate the toxicity of nanomaterials, including rodent, cell culture, zebrafish, and Drosophila system models [72,73,74]. Although the higher-vertebrate platform is an important model for evaluating complicated physiological situations, vertebrates present various disadvantages that make them ill-suited for use in exploring nanotoxicity [75]. The vertebrate animals are costly to obtain, time-consuming to maintain, and may not align with animal welfare concerns. Therefore, cell culture, zebrafish, and Drosophila models have become attractive alternative approaches due to their high throughput and cost efficiency [73,76].

Zebrafish constitute a well-established model that is often applied to study issues of development, disease, and environmental contamination [77,78,79,80]. They exhibit various advantages that make them suitable for studying toxicology [73,81]. Zebrafish are highly efficient models due to their high reproduction rates [81]. Moreover, the maintenance of zebrafish is relatively inexpensive, with small tank requirements, rapid development, and transparent embryos. In addition, the genes of this model organism share 70% similarity with human genes [82,83], and their critical organ systems, such as the nervous system, intestinal system, and cardiovascular systems, are similar those of humans [76,84]. Furthermore, the results of acute toxicity via inhalation or injection in zebrafish have been demonstrated to exhibit a high correlation with zebrafish embryos and rodents. It is worth mentioning that the zebrafish model can help us quickly and efficiently understand cellular and molecular mechanisms. Due to the advancement of genetic tools, mechanisms, such as those of ROS and autophagy triggered by toxicants, can be easily observed via fluorescent reporters and transgenic lines [85,86,87,88].

According to several studies, zebrafish and their embryos have great potential as models to evaluate nanotoxicity, serving as alternatives in the approach for testing nanomaterials [73,76]. Moreover, the zebrafish model provides various means to measure nanotoxicity, such as the quick assessment of productive toxicity, teratogenicity, and developmental toxicity, as well as for evaluating immunotoxicity, genotoxicity, and neurotoxicity [7,89,90,91,92]. For example, zebrafish were employed to evaluate nanoparticle-induced adverse effects. ZnONPs increased the mortality rate of zebrafish embryos and induced malformation phenotypes, such as pericardial edema and yolk-sac edema. Moreover, ZnO affected the expression of inflammatory and immune response genes, including *aicda*, *cyb5d1*, *edar*, *intl2*, *ogfrl2*, and *tnfsf13b* [93]. In addition, silica NPs cause lower blood flow and blood velocity in zebrafish embryos. Silica NPs trigger inflammatory responses via neutrophils and damage vascular endothelial cells [94]. Another study revealed that AgNPs influence the richness and diversity of the microbiota in zebrafish, particularly in males. Therefore, the zebrafish model provides an ideal platform for a relatively quick, high-throughput screening of hazardous nanomaterials and for determining nanomaterial-triggered toxic mechanisms.

As described above, autophagy is an important cellular response induced by nanomaterials. Over the past decade, the zebrafish model gained attention in the autophagy field. Zebrafish, as a tractable animal model, are suitable for use in manipulating the molecular and cellular mechanisms of autophagy [88,95]. Several transgene techniques are applicable to zebrafish models, such as tissue-specific genomic manipulation and the CRISPR system for genome editing. Moreover, several transgenic reporter lines have been used to monitor the autophagy process, including Tg (CMV:EGFP-Mapllc3b) [30], Tg (TαCP:YFP-2XFYVE) [96], and Tg (TαCP:mCherry-GFP-map1 lc3b) [88,95,97]. These techniques promote the understanding of the physiological functions of autophagy in zebrafish. Similarly, the application of these technologies in a zebrafish system to detect nanomaterial-induced autophagy may be an ideal strategy. Indeed, exposure to high doses of TiO_2_NPs has been reported to lead to abnormal testicular morphology and spermatocyte necrosis in zebrafish. The application of TiO_2_NPs resulted in mitochondria being swallowed and autophagic vacuoles accumulating in zebrafish testes [98].

### 4.3. Drosophila Model

*Drosophila melanogaster* has a long history of significant contributions to biomedical research, including in the research of nine Nobel Laureates in Physiology or Medicine. This model organism has orthologs for approximately 75% of human genes and can be profoundly manipulated with genetic and molecular tools. Drosophila also complies with the recommendations of the European Center for the Validation of Alternative Methods (ECVAM), because they present minor practical and ethical obstacles. Further advantages of the fruit fly system includes easy maintenance, low cost, a relatively short life cycle, and much-reduced genetic redundancy compared to mammals, making Drosophila an efficient system for high-throughput screenings and assays [99].

It has been shown that exposure to AgNPs in the diet in an effective Drosophila in vivo platform led to the generation of ROS and high-level autophagy activation, providing strong in vivo evidence that dietary AgNPs activate a series of cytotoxic pathways, including autophagy [45]. In addition, many established Drosophila autophagy transgenic lines are readily available to further study of autophagy induction/activation/dysfunction; for example, UAS-GFP-mCherry-tagged Atg8a is an autophagy reporter transgenic fly line used to monitor the progression of autophagic flux in vivo. These transgenic lines can greatly facilitate the use and development of Drosophila as an in vivo animal model for alternative strategies for testing nanomaterials. Therefore, the Drosophila autophagy system may provide an excellent system for the in vivo assessment of nanoparticle toxicity [100,101]. Furthermore, AgNPs shortened the life span and reduced the stress resistance capacity of the adult flies [45]. Interestingly, other nanomaterials, such as copper oxide nanoparticles (CuONPs), also induced toxicity in Drosophila via ROS; whether CuONPs and other nanomaterials also induce autophagy in Drosophila remains to be explored [102]. As basal autophagy provides a protection mechanism and thus is generally considered a promoter of longevity [103], it is a possibility that AgNPs-induced autophagy activation plays an important role in aging and longevity. Thus, inducing autophagy activation in Drosophila can serve as an alternative testing strategy for determining nanomaterial toxicity. More importantly, autophagy genes, encoding Atg proteins, are structurally, functionally, and mechanistically conserved between Drosophila and humans [104]. 

Nevertheless, the molecular mechanisms by which nanomaterials induce autophagy and shorten the life span of Drosophila remain to be explored. Given the potential risks associated with nanomaterial-induced autophagy in longevity and diseases, it is important to further study autophagy using an in vivo model and to develop systematic alternative strategies for testing nanomaterials, particularly regarding autophagy activation in Drosophila. In other words, Drosophila can serve as a practical and ethical in vivo animal model for alternative strategies for testing nanomaterials and make an important contribution to preventing/treating nanomaterial-induced toxicity in humans. Moreover, it is critical to establish Drosophila autophagy as a systematic and effective alternative strategy for testing nanomaterials for improving nanomaterial risk management and human safety regulations.

### 4.4. Rodent Model

The classic animal models most frequently used in toxicology are mammals, including rodents, dogs, pigs, and non-human primates. However, major species used in nanotoxicology are mainly rodents, such as mice and rats. Laboratory mice are popular research models because of their size, availability, ease of handling, and high genetic similarity to humans. Many mice and rat models are well-established and widely used in pharmacological and toxicological studies of NMs [105,106]. Using mouse models for precisely controlled exposure to NMs, we can perform NMs toxicity assessment include LD50, biodistribution, clearance, hematology, serum chemistry, and histopathology. Biodistribution can evaluate the NMs circulation route and the target organs by measuring fluorescent or radioactive labeled nanoparticles in animals [107]. Examining the excretion and metabolism of nanoparticles at regular time points after exposure can determine the clearance of NMs [108]. Monitoring changes in serum chemistry, cell type, and organ histopathology of mice after nanoparticle exposure is also another method for nanotoxicity assessment [109,110,111]. Coupling with the development of transgenic mice and disease models, we can also take various host factors, such as genetic defects and pre-existing pathology, into consideration in nanosafety [105]. The REACH Guidance states that acute, subchronic, and chronic toxicity, skin and eye irritation, or corrosion and skin sensitization, genetic toxicity, reproductive toxicity, carcinogenicity, and toxicokinetics of NPs should be evaluated when conducting in vivo nanotoxicity assessments. In some cases, REACH requests collection of urine and blood at specified time points, recording the weight of the mice or rats and their behavior, measuring the consumption of food and water [35]. NMs is widely used in multiple medical applications, so the potential side effect of these NMs has become an important issue. As the in vitro and in vivo effects of NPs are not fully matched, side effects cannot be accurately estimated by in vitro tests, it is recognized that animal tests are essential for safety assessment of these medical NMs [106]. For example, AgNPs is one of the most common NMs in medical use because of its antimicrobial activity. Of course, many studies have evaluated the safety and biocompatibility of AgNPs in rodent models. Kim et al. have reported the results of a 28-day oral exposure study of 60 nm AgNPs in rats [112]. Data showed dose-dependent changes in serum cholesterol and ALP and liver damage after 300 mg AgNPs treatment. In addition, eosinophil infiltration of the hepatic lobules and portal tract and bile duct hyperplasia was also found. Another subchronic dermal toxicity assessment analyzed biodistribution of AgNPs in guinea pigs, and the results showed that the tissue level of AgNPs and dermal exposure show a dose-dependent correlation with the following ranking: kidney > muscle > bone > skin > liver > heart > spleen [113]. Histopathological data further reported toxicities in kidney, bone, and cardiocytes after dermal exposure to AgNPs. Chuang’s group also established allergen-provocation mice models to investigate the effects of inhaled AgNPs in healthy and allergic individuals [114]. 

## 5. Autophagy Detection as a Toxicity Biomarker-Like Indicator for Medical, Food, and Cosmetic NPs Safety Assessments in Future

### 5.1. Silver Nanoparticles-Induced Toxicity and the Possible Role of Autophagy

Silver has been used in our lives throughout history and recently for many medical applications due to its effectiveness in arresting the growth of microorganisms [115]. Recent studies have shown the enormous therapeutic potential of AgNPs against numerous cancer cells by modulating autophagy action as cytotoxic agents or as nanocarriers that, combined with other treatments, deliver therapeutic molecules [116,117]. The toxicity of AgNPs has been suggested as the result of lysosome-dependent silver ion release that leads to massive ROS production. These ROS cause disruption of the lysosomal membrane integrity and enables the escape of AgNPs into the cytosolic space, through which they subsequently target other subcellular compartments [118]. In addition, AgNPs-induced lysosomal dysfunction, including loss of membrane integrity or internal acidity, is also related to an impaired autophagosome–lysosome fusion process that critically interferes with the functionality of the autophagy machinery [119]. AgNPs have high affinity for thiol groups, which are important for protein folding and function as ROS scavengers. Therefore, AgNPs cause the protein misfolding that induces the ER stress and glutathione depletion that leads to ROS metabolism imbalance. All of these results enhance the autophagy process and ultimately cause cell death.

It has been documented that AgNPs are potential sources of oxidative stress, leading to ROS production and subsequent autophagy induction in the NIH3T3 mouse embryonic fibroblast cells to which they were exposed [18]. In another study, administration of AgNPs upregulated LC3-II protein expression and accumulated in liver tissue [120]. Under oxidative stress conditions, autophagy can be induced to suppress cellular ROS levels in a cell survival mechanism of normal cells. While it has also been reported that chronic low-dose AgNPs exposure resulted in to HaCaT noncancerous cell transformation, and despite the activation of EGF receptors and the related gene expression that enhances cell proliferation, cells treated with a high dose of AgNPs within a short time showed inhibited proliferation [121]. Ag-NPs have been observed to have a higher cytotoxic effect on PANC1 pancreatic cancer cells than on non-tumor cells of the same tissue [122]. Furthermore, combining AgNPs with drugs synergistically enhanced the cytotoxicity to cancer cells [116]. In addition to autophagy induction, AgNPs have also been demonstrated to block autophagic flux to induce autophagosome accumulation, resulting in the impedance of monocyte–macrophage differentiation [123]. These findings provide new perspectives on anticancer therapy strategies using nanomaterials.

### 5.2. ZnO Nanoparticles-Induced Toxicity and the Possible Role of Autophagy

ZnONPs have been employed in biomedical and cancer applications due to their unique properties [124]. It has been reported that ZnONPs induced significant cytotoxicity with increased intracellular ROS and oxidative stress that led to apoptosis and autophagy in SKOV3 ovarian cancer cells [125]. ZnONPs have also been observed to induce toxicity by activating PINK1/Parkin-mediated mitophagy in CAL27 oral cancer cell lines [126]. In addition, some studies reported that ZnONPs exhibited preferential cytotoxicity to highly proliferative tumor cells through a lysosome-mediated zinc ion release mechanism that subsequently led to ROS-mediated cell death [124,127]. These reports strongly suggest the potential of ZnONPs as anticancer agents.

In the cosmetics industry, ZnONPs are present in daily supplies, such as shampoos, conditioners, soaps, deodorants, sunscreens, and skin care products, and makeup, in general, to function as antibacterial agents, UV-filters, and pigments and for deeper skin penetration, anti-wrinkling, or moisturizing [128,129,130]. For example, sunscreens containing ZnONPs and TiO_2_NPs are effective barriers against ultraviolet light (UV-light) damage to skin and do not leave white or other residues on the skin [131,132]. AgNPs are used in toothpaste and soap and other cleaning products to achieve an antibacterial effect, and gold nanoparticles (AuNPs) are commonly used as carriers that easily penetrate the skin [132,133,134]. However, the diversity of NMs applied in cosmetics has raised concerns about their potential risks. Studies have indicated that NMs can be translocated to main organs, such as the brain, kidney, or heart, from different exposure routes; the transdermal penetration and translocation of NMs through the skin are still controversial [135,136,137]. Zvyagin et al. reported that ZnONPs stayed in the stratum corneum (SC) and accumulated into skin folds and/or hair follicle roots when applied topically in excised and in vivo human skin. [137]. In contrast, skin exposure to ZnONPs and TiO_2_NPs led to the incorporation of nanoparticles in the SC and induced phototoxicity and genotoxicity [138]. Numerous studies have reported the autophagy-inducing activities of ZnONPs. For example, abnormal autophagosome accumulation and mitochondrial dysfunction was observed in normal ZnONPs-treated skin cells, and ZnONPs toxicity was found to be related to the induction of ROS in a concentration- and time-dependent manner [139]. In another study, ZnONPs induced ROS generation in immune cells and activated autophagy through PI3K/Akt/mTOR signaling pathway inhibition [140]. All these results show the ROS-related autophagy-inducing and cytotoxicity-inducing abilities of ZnONPs. Therefore, the toxicity of ZnONPs to cells has attracted researchers’ attention. 

### 5.3. TiO_2_ Nanoparticles-Induced Toxicity and the Possible Role of Autophagy

TiO_2_NPs, which also serve as common ingredients in sunscreens and cosmetics, through which they absorb ultraviolet radiation, have also been investigated as autophagy modulators [141]. TiO_2_NPs induced autophagy in primary human keratinocytes in a dose-dependent manner, which played a vital role in determining keratinocyte survival [142]. Another study also revealed that TiO_2_NPs induced autophagy at a low dose while blocking autophagic flux at a high dose, which is caused by a large amount of TiO_2_ NP accumulation-mediated overload of the degradative capacity of human keratinocytes [143]. Generally, TiO_2_NPs induce lower toxicity in cells than ZnONPs because of their resistance to lysosome degradation and low metal ion release [139]. These findings suggest that a pro-survival mode of autophagy induction by TiO_2_NPs provides further insights into the debate of the NPs for use in consumer products.

Recently, the unique super-photocatalytic properties of TiO_2_NPs showed potential application in photodynamic therapy (PDT) upon irradiation [144]. It has been reported that TiO_2_NPs were successfully used in PDT for many different types of cancers [145,146]. Under UV light illumination, the excited valence band electrons in TiO_2_ jump to the conduction band, resulting in electron holes that have the ability to generate various ROS, including hydroxyl radicals (OH·), hydrogen peroxide (H_2_O_2_), and superoxide (O_2_^−^) [145]. Excess ROS can further trigger autophagy-associated apoptotic cell death, making TiO_2_ much more efficient at killing cancer cells. These findings provide another application potential of TiO_2_NPs as anticancer agents.

In food industry, most of the nanoparticle in the products are designed “out-of-food” that are not directly added to human food but some of these products have been used as food pigments and colorants [147]. For instance, inorganic oxide chemicals such as SiO_2_ (E551), MgO (E530), and TiO_2_ (E171) are used as anti-caking agents, food flavor carriers, food pigments, and colorants that are permitted by the U.S. FDA [147]. Food-grade TiO_2_NPs are the most widely used NPs in food, as additives in gum, white sauce, cake icing, candy, and pudding, with approximately 40% at concentrations in the nanometer range [148]. TiO_2_NPs, AgNPs, and ZnONPs have also been used in food packaging because each can be easily and effectively incorporated into nanocomposites to inhibit bacterial growth and extend the shelf life of food products [149]. As mentioned above, NPs might increase the levels of intracellular ROS, in turn damaging mitochondria and the ER, leading to apoptosis, DNA damage, an impaired cell cycle, and autophagy [148,150]. However, ROS production may not be the sole mechanism for the toxicity found in vitro with NPs. Previous studies have indicated that TiO_2_NPs may interact with DNA directly, since particles were detected by transmission electron microscopy inside the nucleus in various cells, including blood lymphocytes and nasal, pulmonary, and dermal cells [151]. TiO_2_NPs-triggered DNA damage can also be induced through indirect mechanisms, such as dysregulated cell division, DNA replication, transcription, and repair [151]. Zhang et al. indicated that TiO_2_NPs entered trophoblast HTR-8/SVneo cells and were distributed primarily to lysosomes, where they ultimately induced autophagy in the cells [152]. Thus, food NPs could trigger autophagy blockage and lead to the accumulation of damaged ER and mitochondria and the production of ROS, resulting in further cellular damage such as NLRP3 inflammasome activation [51]. These findings provide insight into autophagy and may account for the early and specific toxicity-inducing mechanisms of NPs.

## 6. Conclusions and Perspectives

**Figure 2:**
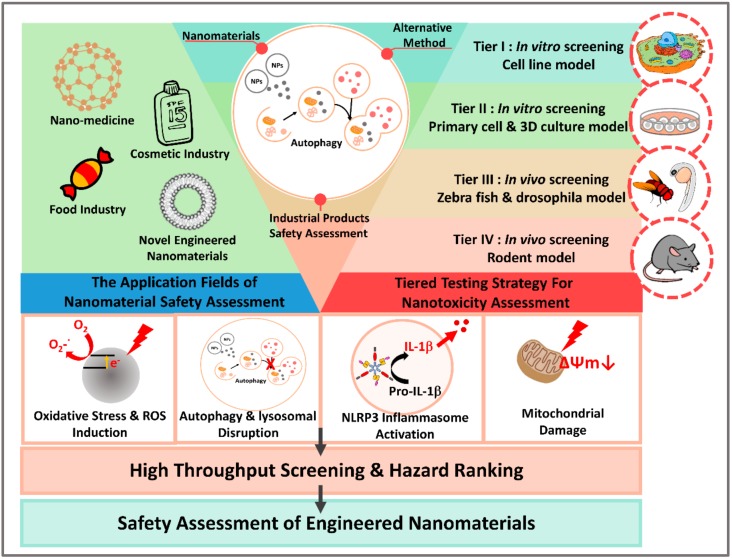
The proposed predictive, tiered toxicological testing strategy for nanomaterial hazard testing. The tiered testing strategy we have suggested for the evaluation of the toxicity of nanomaterials is based on screening with in vitro cell lines and high-throughput systems (Tier I). The next testing step is performed using primary cells and a 3D cell culture system to increase confidence in the data obtained from the cell lines (Tier II). Then, the zebrafish and/or Drosophila models (Tier III) are used to fill the gap in the in vitro, and the potential effects are then detected in rodents (Tier IV). When a significant potential hazard is identified in these test system steps, the rodent toxicity testing is needed. More importantly, we focused on the assessment of autophagy-related effects (autophagy and lysosomal dysfunction) and oxidative stress-related responses (ROS, mitochondrial damage, and DNA damage) as primary sensitive markers for evaluating the toxicity of the nanomaterials. As autophagy is a significant and sensitive effect induced by nanomaterials, evaluating autophagy-related pathways in the first step would improve the testing efficiency of the nanomaterials. The development of a tiered testing strategy for nanomaterial (NM) hazard assessment not only promotes the widespread adoption of non-rodent models and/or the 3Rs (reduces, refines, or replaces) principle but also makes nanotoxicology testing more ethical, relevant, and cost- and time-efficient.

Nanotechnology has potential to be widely utilized in different fields, including the pharmaceutical, food, and cosmetics industries. The biosafety issue of nanoparticles has drawn great attention because many NPs induce various levels of cytotoxicity that eventually lead to cell death, cell cycle arrest, or differentiation disruption. It should also be noted that the toxicity induced by the long-term exposure to NPs has elicited significant concerns, especially the toxic effects to fertility, carcinogenesis, neuron, skin, and the gastrointestinal tract [138,148,153,154,155]. All these concerns suggest that more research and optimized evaluation systems are needed to define the exact mode of toxicity of NPs. Additionally, uncovering the underlying mechanisms that regulate toxicity will contribute greatly to adequate hazard assessments of NPs and regulation and legislation development for the management of NPs. Although several in vitro toxicity tests or in silica analyses covering important toxicity endpoints have been established as high-throughput methods for the evaluation of chemical toxicity [8,22,24,26], universally accepted protocols and well-designed alternative testing strategies for NP toxicity are still relatively scarce. A primary cause of these limited protocols and strategies is the complexity of the physicochemical properties of NPs and of NM interactions with biological systems that dictate the diverse fates of exposed cells. To fill the gaps of understanding on nano–bio interactions, more systematic research approaches using high-throughput in vitro models are needed to provide toxicity results of NM use (Figure 2). Of importance, a better understanding of the interaction of NPs with cells, tissues, and organs, for addressing critical issues related to toxicity testing, especially with respect to alternatives to tests on animals, is needed. While nanotoxicity is often the major concern when toxicologists discuss the novel nanomaterials’ safe assessment, there are many nanomaterials that have been applied for therapeutic or industry purposes. Since the introduction of the first FDA-approved nano-drug in 1995, nanomedicine has constantly revolutionized medical therapeutics and diagnostics [156]. Manipulating molecules and atoms in the nanoscale has empowered researchers to come up with novel particles and formulations that possess more beneficiary characteristics and less unwanted features [156].

Adverse outcome pathways (AOPs) are an important tool to organize data and facilitate the understanding of the specific bioactivity of NPs. With respect to AOPs, this review demonstrated that autophagy and ROS production elicited by NPs appeared to be critical responses to toxicity, as autophagy is a basic stress response and a potential regulator of toxicity. Undoubtedly, the autophagic effects of NPs are highly dependent on their physicochemical characterization [51]. Some NPs can induce both autophagy blockage and autophagic flux in different testing systems. Autophagy dysfunction can lead to the accumulation of damaged DNA, proteins, and organelles that in turn increase the risk of cancer, neurodegenerative diseases, and reproductive dysfunction [51]. Effective risk assessment of NPs depends on in vitro testing strategies and relevant non-mammalian models with sufficient sensitivity to these substances. The integrated approach applying autophagy as an early sensitivity marker combined with the appropriate AOPs would enable the determination of the possible toxicity of NPs. In this review, we focused on NPs that are widely used in several industries, describing their applications, toxic effects, and autophagy-inducing potency. We also discussed several alternative methods for nanoparticle toxicity evaluation and suggested the potential application of autophagy as a tier I early toxicity endpoint in the testing framework. These results will enable the development of more-relevant testing strategies to predict the possible long-term toxicity of NPs. In addition, these strategies can be applied in the future for regulatory decision making and risk assessment of NP uses (Figure 2).

## Abbreviations

3R’s	Reduces, Refines, or Replaces
ACD	Autophagic Cell Death
AgNPs	Silver Nanoparticles
AMPK	AMP Kinase
AOP	Adverse Outcome Pathway
AuNPs	Gold Nanoparticles
BBB	Blood–Brain Barrier
BPR	Biocidal Products Regulations
CLP	Classification, Labelling and Packaging
CuONPs	Copper Oxide Nanoparticles
DMH	Dimethylhydrazine
ECVAM	European Center for the Validation of Alternative Methods
ER	Endoplasmic Reticulum
FADD	Fas-Associated Protein with Death Domain
GIT	Gastrointestinal Tract
INRA	French National Institute for Agricultural Research
ISO	International Organization for Standardization
LMP	Lysosomal Membrane Permeabilization
NCI	National Cancer Institute
NCL	Nanotechnology Characterization Lab
NMs	Nanomaterials
NRC	National Research Council
OECD	Organization for Economic Co-operation and Development
PDT	Photodynamic Therapy
REACH	Registration, Evaluation, Authorization and Restriction of Chemicals
ROS	Reactive Oxygen Species
SC	Stratum Corneum
SCCS	Scientific Committee on Consumer Safety
SEURAT	Safety Evaluation Ultimately Replacing Animal Testing
TEM	Transmission Electron Microscopy
TiO_2_NPs	Titanium Dioxide Nanoparticles
TT21C	Toxicity Testing in the 21st Century
UV	Ultraviolet
UV-light	Ultraviolet Light
ZnONPs	Zinc Oxide Nanoparticles
